# Supplementary material for: Modeling glioblastoma heterogeneity as a dynamic network of cell states
Source: Mol Syst Biol. 2021 Sep 16;17(9):e10105. doi: 10.15252/msb.202010105 (PMC8444284; doi:10.15252/msb.202010105)
Supplement: Supplementary file 6 — Source Data for Figure 5 [file MSB-17-e10105-s004.zip › Figure5A_sourcedata/GSEA_3017/nosigdb_stateA.GseaPreranked.1622795026485/DOROSZKO_U3180MG_CLUSTER_2.html]

Details for gene set DOROSZKO\_U3180MG\_CLUSTER\_2[GSEA]

|  || Dataset | state53017 |
| Phenotype | NoPhenotypeAvailable |
| Upregulated in class | na\_neg |
| GeneSet | DOROSZKO\_U3180MG\_CLUSTER\_2 |
| Enrichment Score (ES) | -0.47268394 |
| Normalized Enrichment Score (NES) | -2.7423663 |
| Nominal p-value | 0.0 |
| FDR q-value | 0.0 |
| FWER p-Value | 0.0 |
Table: GSEA Results Summary

  

Fig 1: Enrichment plot: DOROSZKO\_U3180MG\_CLUSTER\_2      
 Profile of the Running ES Score & Positions of GeneSet Members on the Rank Ordered List

  

| PROBE | GENE SYMBOL | GENE\_TITLE | RANK IN GENE LIST | RANK METRIC SCORE | RUNNING ES | CORE ENRICHMENT || 1 | IGFBP3 |  |  | 26 | 0.641 | -0.0090 | No |
| 2 | EPN2 |  |  | 41 | 0.582 | -0.0067 | No |
| 3 | JMJD1C |  |  | 81 | 0.470 | -0.0350 | No |
| 4 | YME1L1 |  |  | 145 | 0.412 | -0.0911 | No |
| 5 | TNC |  |  | 159 | 0.402 | -0.0931 | No |
| 6 | MYC |  |  | 169 | 0.395 | -0.0910 | No |
| 7 | TUBGCP2 |  |  | 196 | 0.378 | -0.1079 | No |
| 8 | ITGB1 |  |  | 256 | 0.346 | -0.1617 | No |
| 9 | GALR1 |  |  | 267 | 0.340 | -0.1624 | No |
| 10 | HSPD1 |  |  | 291 | 0.328 | -0.1775 | No |
| 11 | CREB5 |  |  | 407 | 0.291 | -0.2939 | No |
| 12 | PGP |  |  | 503 | 0.270 | -0.3892 | No |
| 13 | SRSF2 |  |  | 542 | 0.262 | -0.4226 | No |
| 14 | ZNF37A |  |  | 571 | 0.256 | -0.4454 | No |
| 15 | AGT |  |  | 587 | 0.252 | -0.4541 | No |
| 16 | ITGAV |  |  | 599 | -0.250 | -0.4585 | No |
| 17 | GOLGB1 |  |  | 605 | -0.253 | -0.4564 | No |
| 18 | AIG1 |  |  | 621 | -0.260 | -0.4649 | Yes |
| 19 | GPR37L1 |  |  | 624 | -0.261 | -0.4592 | Yes |
| 20 | VMP1 |  |  | 632 | -0.262 | -0.4589 | Yes |
| 21 | CAMK2D |  |  | 635 | -0.264 | -0.4531 | Yes |
| 22 | SDC3 |  |  | 637 | -0.264 | -0.4463 | Yes |
| 23 | MAGI1 |  |  | 642 | -0.266 | -0.4426 | Yes |
| 24 | GCC2 |  |  | 661 | -0.276 | -0.4539 | Yes |
| 25 | RAB3IP |  |  | 667 | -0.280 | -0.4509 | Yes |
| 26 | CHD9 |  |  | 673 | -0.284 | -0.4478 | Yes |
| 27 | DDR1 |  |  | 683 | -0.294 | -0.4487 | Yes |
| 28 | TMEM59L |  |  | 698 | -0.299 | -0.4549 | Yes |
| 29 | SERINC1 |  |  | 701 | -0.302 | -0.4480 | Yes |
| 30 | ATP6AP2 |  |  | 705 | -0.304 | -0.4421 | Yes |
| 31 | ZEB2 |  |  | 722 | -0.319 | -0.4499 | Yes |
| 32 | BICD1 |  |  | 723 | -0.319 | -0.4403 | Yes |
| 33 | SPTBN1 |  |  | 725 | -0.322 | -0.4317 | Yes |
| 34 | HES1 |  |  | 736 | -0.331 | -0.4326 | Yes |
| 35 | JAG1 |  |  | 745 | -0.339 | -0.4311 | Yes |
| 36 | IAH1 |  |  | 749 | -0.343 | -0.4240 | Yes |
| 37 | MYO6 |  |  | 750 | -0.343 | -0.4137 | Yes |
| 38 | MEIS2 |  |  | 754 | -0.345 | -0.4066 | Yes |
| 39 | NTM |  |  | 762 | -0.354 | -0.4035 | Yes |
| 40 | MSI2 |  |  | 771 | -0.361 | -0.4013 | Yes |
| 41 | ITGB8 |  |  | 774 | -0.363 | -0.3926 | Yes |
| 42 | SNTG1 |  |  | 777 | -0.367 | -0.3837 | Yes |
| 43 | IRS2 |  |  | 781 | -0.368 | -0.3759 | Yes |
| 44 | MACF1 |  |  | 785 | -0.371 | -0.3680 | Yes |
| 45 | APP |  |  | 812 | -0.394 | -0.3844 | Yes |
| 46 | DST |  |  | 818 | -0.400 | -0.3778 | Yes |
| 47 | RTN4 |  |  | 837 | -0.429 | -0.3845 | Yes |
| 48 | CPEB4 |  |  | 857 | -0.447 | -0.3917 | Yes |
| 49 | DCLK2 |  |  | 859 | -0.449 | -0.3792 | Yes |
| 50 | SLC1A2 |  |  | 860 | -0.450 | -0.3657 | Yes |
| 51 | AKAP9 |  |  | 869 | -0.476 | -0.3600 | Yes |
| 52 | SLC35F1 |  |  | 871 | -0.482 | -0.3466 | Yes |
| 53 | RGS12 |  |  | 880 | -0.504 | -0.3401 | Yes |
| 54 | DAAM1 |  |  | 885 | -0.516 | -0.3289 | Yes |
| 55 | F3 |  |  | 888 | -0.526 | -0.3153 | Yes |
| 56 | CALD1 |  |  | 889 | -0.527 | -0.2994 | Yes |
| 57 | MEG3 |  |  | 894 | -0.537 | -0.2876 | Yes |
| 58 | SAT1 |  |  | 909 | -0.573 | -0.2855 | Yes |
| 59 | PTX3 |  |  | 919 | -0.603 | -0.2772 | Yes |
| 60 | CLU |  |  | 938 | -0.685 | -0.2761 | Yes |
| 61 | HEPACAM |  |  | 943 | -0.709 | -0.2591 | Yes |
| 62 | CDH2 |  |  | 947 | -0.762 | -0.2394 | Yes |
| 63 | PPP1CB |  |  | 962 | -0.885 | -0.2280 | Yes |
| 64 | ZBTB20 |  |  | 964 | -0.895 | -0.2021 | Yes |
| 65 | SLC1A3 |  |  | 973 | -1.097 | -0.1778 | Yes |
| 66 | HEPN1 |  |  | 975 | -1.110 | -0.1454 | Yes |
| 67 | CYR61 |  |  | 978 | -1.296 | -0.1086 | Yes |
| 68 | SPARCL1 |  |  | 984 | -1.810 | -0.0595 | Yes |
| 69 | NEAT1 |  |  | 986 | -2.049 | 0.0011 | Yes |
Table: GSEA details [plain text format]

  

Fig 2: DOROSZKO\_U3180MG\_CLUSTER\_2: Random ES distribution      
 Gene set null distribution of ES for **DOROSZKO\_U3180MG\_CLUSTER\_2**
